# Mind the Gender and Generation Gaps - How the Drivers of Population Change Affect Democracy

**DOI:** 10.1093/geroni/igaf122.1261

**Published:** 2025-12-31

**Authors:** Gemma Carney

**Affiliations:** Queen’s University Belfast, Belfast, Northern Ireland, United Kingdom

## Abstract

Political participation of older people has not been extensively researched in gerontology. Nor has age been a concern of political scientists. The resulting gap in the literature has been filled by supposition, such as the claim that all Trump voters are aged. These conspiracy theories proliferate during a period of intense political polarization, often referred to as ‘the culture wars.’ The culture wars focus on identity, but age has not emerged as an identity with an accompanying political movement like class or gender. As a result, democracies with older populations lack policies appropriate to an ageing population despite an active, older electorate. Yet older voters are influencing electoral outcomes. Cultural Backlash theory (Norris and Inglehart, 2019) makes an empirical link between the ageing of the electorate and the rise of socially conservative leaders. In this paper, I set out a research agenda which rises to broader, long-term challenges for democracy in ageing societies. I outline the importance of political demography, which analyses the political implications of population ageing, for gerontology. I argue that the drivers of population change – fertility, migration and extended life expectancy - all have important implications for democracy. We can see this in the pro-natalist ideology of Italy’s Prime Minister Giorgia Meloni and in the success of anti-migrant populist parties around the world. Most urgently, we must pay urgent attention to who is deciding not to vote, namely women and younger cohorts. The paper concludes that a focus on political demography in gerontology is urgently needed.

